# Interprofessional education program: perceptions and experiences among Brazilian participants

**DOI:** 10.1186/s12909-026-08810-x

**Published:** 2026-02-24

**Authors:** Marcela Pereira Oliveira, Renata Kobayashi, Milton Arruda Martins, Patrícia Zen Tempski, Fernanda Magalhães Arantes-Costa

**Affiliations:** 1 University Center of Valença, R. Sargento Vitor Hugo, N° 161, Fátima, Valença, RJ CEP: 27603-086 Brazil; 2 Center for the Development of Medical Education, School of Medicine, University of São Paulo, Av. Dr. Arnaldo, 455, Room 2349, São Paulo - SP, 01246-903 Brazil; 3 Conscientia, São Leopoldo Mandic, R. Dr. José Rocha Junqueira, 13, Campinas, São Paulo, SP 13045-755 Brazil

**Keywords:** Interprofessional education, Interprofessional teams, Community-based interprofessional education

## Abstract

**Background:**

Interprofessional education (IPE) is fundamental to developing collaborative competencies and, consequently, effective teamwork. The Health Work Education Program (PET-Health) is a Brazilian federal initiative that promotes interprofessional and collaborative practices within the community. Its aim is to strengthen the teaching–service–community relationship, qualify professionals working in the Unified Health System, and support student training to address community health issues. The 9th PET-Health call for proposals selected programs that foster the teaching–service–community relationship through interprofessional initiatives. The proposal submitted by the University Center of Valença was approved, and activities began in April 2019. This study aimed to assess the impact of the PET-Health program on the development of collaborative competencies through a qualitative investigation. The study is justified by the need to ensure coordinated, safe, and effective care that meets patients’ needs.

**Methods:**

This exploratory qualitative study was based on narratives from students and preceptors participating in an interprofessional PET-Health program at the University Center of Valença (UNIFAA). Narratives were analyzed using the software *Interface de R pour les Analyses Multidimensionnelles de Textes et de Questionnaires* (IRaMuTeQ), version 0.7 alpha 2. The methodological approach was grounded in a phenomenological perspective, allowing participants’ perceptions to emerge authentically and without distortion.

**Results:**

The corpus comprised 25 narratives, divided into 321 text segments (TSs), of which 272 (84.74%) were retained for analysis. A total of 11,382 occurrences (words or forms) and four classes emerged. Class 1 highlighted the program’s role in clarifying professional roles, reducing stereotypes, and strengthening communication. Class 2 addressed the challenges imposed by the COVID-19 pandemic. Class 3 described adaptations, such as remote health education, implemented to ensure program continuity. Class 4 emphasized the value of community-based teaching in fostering collaborative competencies and the preceptor’s role as a mediator in this context.

**Conclusions:**

The PET-Health program fosters teamwork. Participants reported improved communication with other professionals, reduced stereotypes, and a deeper understanding of the roles of different healthcare workers. Although the COVID-19 pandemic hindered in-person activities, it stimulated the use of remote strategies and information and communication technologies in health education. Preceptors play a central role in integrating teaching, service, and community, as well as fostering the development of collaborative competencies. Participants emphasized the importance of implementing interprofessional education in both student training and professional development. Longitudinal studies are needed to assess the impact of IPE on population health outcomes. Interprofessional education is fundamental to training health professionals and should be embedded in curriculum guidelines, pedagogical programs, and public health policies.

**Supplementary Information:**

The online version contains supplementary material available at 10.1186/s12909-026-08810-x.

## Background

There are different health system models around the world, some of which are fragmented and unable to meet society’s healthcare demands. This issue is exacerbated by the shortage of health professionals, their unequal distribution, and inadequate training [1].

Patient safety remains a critical and recurring concern, revealing significant challenges in the education and training of healthcare professionals. Research consistently highlights that insufficient collaboration—particularly deficiencies in communication—is a key factor contributing to patient safety issues.

The landmark study *To Err Is Human*, published by the Institute of Medicine, estimated that between 44,000 and 98,000 deaths occur annually in the United States as a result of preventable medical errors. Ineffective communication was identified as one of the primary contributing factors [2]. Data from the Joint Commission (2012) also indicate that communication failure is among the root causes of more than 70% of adverse events in healthcare delivery [3].

Two decades after its publication, many improvements are still needed in health systems. These include enhancing outpatient care, improving the use of health information and communication technologies, safeguarding patient data in electronic medical records, reducing diagnostic errors, and—above all—strengthening teamwork and ensuring patient safety [4].

Given the significant challenges facing healthcare, and the growing need to strengthen the workforce, improve the education and training of healthcare professionals, and prioritize patient safety, the World Health Organization (WHO) launched a document encouraging countries to adopt interprofessional education (IPE) in the training of health professionals and students [1].

IPE occurs when two or more professionals learn with, from, and about each other to collaborate and deliver quality healthcare (Advanced Center for Interprofessional Education) [5]. Originally defined in the United Kingdom, this concept is now widely adopted globally. Its core message is: “learn together to work together” [5].

IPE has been widely adopted in community healthcare for both vulnerable groups and in chronic and acute care settings. Rehabilitation, ensuring safety, facilitating discharge, and maintaining quality of life are key goals emphasized in IPE [5].

To improve population health outcomes as well as the quality of individual care, the United States introduced the *Core Competencies for Interprofessional Collaborative Practice*. These competencies are based on the premise that interprofessional collaborative practice leads to improved healthcare delivery [6].

For health outcomes to improve and for professionals to collaborate effectively, opportunities must be provided for different professionals to learn about, from, and with each other [1]. This learning should not be limited to sharing classroom spaces, wards, or primary care units. Instead, it should emphasize student interaction as a foundation for learning [7].

Despite a decade of encouragement to implement IPE, there is limited evidence of changes in professional practice or improvements in population health outcomes [8]. Selecting appropriate learning methods is essential for the successful implementation of IPE. The literature highlights several approaches, including seminar-based learning, observation-based learning, problem-based learning, simulation-based learning, clinical practice-based learning, e-learning, and blended learning [9].

One initiative to promote IPE was the PET-Health Program, launched in 2010 by the Ministry of Health. The program aims to strengthen the relationship between teaching, service, and community; enhance the qualifications of professionals within the Unified Health System; and prepare students to address community health needs [10].

In 2017, Brazil, stimulated by the Pan American Health Organization, included interprofessional education as one of its strategies for strengthening professional training in health. Coordination was promoted between the Ministry of Education, Higher Education Institutions, and the Brazilian Network for Interprofessional Education and Work in Health (ReBETIS), which developed a national plan comprising five lines of action: strengthening IPE as a mechanism for reorienting undergraduate health courses; surveying IPE initiatives in Brazil; promoting faculty development for IPE; enhancing spaces for disseminating and producing IPE-related knowledge; and integrating IPE into Permanent Health Education settings [11].

The 9th PET-Health call for proposals sought to select programs that promote teaching–service–community integration through interprofessional initiatives. The proposal submitted by the University Center of Valença was approved, and activities began in Brazil in April 2019 [12].

The UNIFAA PET-Health/Interprofessionality group comprises four tutorial groups including teachers, preceptors, and students from the Nursing, Physical Education, Medicine, Dentistry, and Psychology programs. The program focused on chronic noncommunicable diseases. Each tutorial group developed activities on the promotion, prevention, and rehabilitation of chronic noncommunicable diseases in collaboration with family health teams within their respective territories. Among the assumptions for the development of the program was the intentional formation of interprofessional groups.

The aim of this study was to assess the impact of the PET-Health Program on the development of collaborative competencies through a qualitative investigation. This study is justified by the need to ensure care that is coordinated, safe, and effective, meeting patients’ needs. It also seeks to support the objectives outlined in the WHO document and reinforce the strategic lines of action described in the IPE development plan by the Pan American Health Organization.

The choice to evaluate the PET-Health program is further justified by the fact that the project incorporates interprofessional education into the Brazilian Unified Health System (SUS), promotes the integration of teaching, service, and community, and aligns with global health education policies.

## Methods

This exploratory qualitative study was based on narratives from students and preceptors participating in an interprofessional PET-Health program at the Valença University Center (UNIFAA). The research was approved by the ethics committee of the University Center of Valença (protocol: 33,711,620.2.0000.5246).

The program began in April 2019 and ended in March 2021. The UNIFAA PET-Health/Interprofessional program included four groups, each composed of two teachers, four preceptors, and six students from nursing, physical education, medicine, dentistry, nutrition, and psychology, totaling 48 participants.

Over two years, the program developed activities such as diagnosing community health in relation to chronic noncommunicable diseases; surveying community problems; planning health actions; implementing interventions with health teams and the community; and evaluating these actions. Based on the activities carried out in the community, the groups were also guided to produce scientific evidence.

A blended learning methodology was adopted. The tutorial groups initially diagnosed community health using problem-based learning and subsequently carried out health interventions.

### Data collection

Participation in the study was voluntary, and invitations were sent via email. Inclusion criteria comprised students and preceptors involved in the interprofessional PET-Health program at the University Center of Valença. Exclusion criteria included participants who withdrew or left the program before its conclusion.

Narratives were collected through Google Forms. The data were anonymized, and each participant was assigned a number instead of their name. To submit their narrative, participants first had to agree to the terms of the informed consent form.

Narratives can be understood as fictional or real accounts told or retold [13]. They were collected after the end of the program, and participants had one month to submit them through the electronic form. The timing of collection aimed to allow participants to share all experiences and challenges encountered during the program.

Narratives were chosen for the richness of the information obtained. They enabled understanding of the details and complexities of the participants’ experiences throughout the program. The collection also occurred during the COVID-19 pandemic, a period marked by social distancing.

The purpose of using narratives was to understand participants’ perceptions and reactions to IPE, as well as to identify perceived gains, the development of skills and attitudes, and the limitations encountered in implementing IPE at this educational institution.

The question used in the study was developed specifically for this research. The guiding question for the narrative was:

*Considering the lessons learned, limitations encountered, and observed changes in behavior, how are you currently applying, and how do you plan to apply, the skills acquired in the program to your practice?*

### Data analysis

We used the COREQ model to assess the quality of the research process and data analysis. The checklist consists of 32 items covering the following domains: research team and reflexivity, study design, data analysis, and reporting of findings. The instrument served as a guide for both the planning and development of the manuscript, supporting a systematic approach to qualitative research [14]. The completed COREQ checklist is provided in Supplementary File S1.

The narratives were analyzed using the software *Interface de R pour les Analyses Multidimensionnelles de Textes et de Questionnaires* (IRaMuTeQ), version 0.7 alpha 2. The purpose of using this software was to identify lexical patterns that reflect the semantic structure of lived experience, thereby supporting the emergence of participants’ perceptions.

The main objective of the software is to analyze the structure and organization of discourse to determine relationships between the lexical worlds most frequently described by research participants. IRaMuTeQ is a free tool that operates through Python and R to analyze a collection of texts compiled into a corpus. It enabled the triangulation of lexical and thematic analysis, enhancing the coherence and rigor of the findings [15].

Descending Hierarchical Classification (CHD) was applied. In addition to providing lexical analysis of the corpus, CHD offers contextual lexical classes characterized by specific vocabulary and text segments that share this vocabulary. Using the software, CHD was performed to construct a dendrogram with emerging classes. The higher the χ^2^ value, the more strongly the word was associated with the class, while words with χ^2^ < 3.80 (p < 0.05) were disregarded. The chi-square test is a built-in configuration of IRaMuTeQ, and we chose not to modify it. Importantly, χ^2^ values were not used for direct statistical analysis of the narratives; rather, they served solely as a technical criterion of the software to indicate the strength of association between words and classes [15].

Merleau-Ponty’s phenomenology of perception was adopted as the theoretical framework, guiding all stages of the study and serving as the lens through which participants’ narratives were understood. Perception is not concrete, fixed, or objective, but rather dynamic and shaped by context, interactions, and emotions [16]. This theoretical perspective was combined with lexical analysis using IRaMuTeQ, through which textual patterns were identified and interpreted based on participants’ experiences. Although the study draws on Merleau-Ponty’s phenomenological approach, it does not constitute a phenomenological study. This approach was used to provide a perspective for understanding the lived experiences of participants in the PET-Health program.

For data analysis, the first step was to prepare the text corpus according to the software’s requirements. This preparation included correcting spelling and punctuation and standardizing the document. The second step consisted of the analysis using Descending Hierarchical Classification (CHD). Based on the IRaMuTeQ analysis, lexical classes were identified, and multiple interpretive codes were developed from the concepts and most frequent words emerging from participants’ narratives. In the final stage, the results were systematized and interpreted in light of the literature. Each class was discussed based on the most frequent words generated by CHD, and classes were titled according to the themes corresponding to the text segments.

To increase the validity of the findings, the three researchers employed triangulation to minimize individual bias. All researchers read the narratives and the analyses produced by IRaMuTeQ and reached a final consensus.

To reduce the risk of social desirability bias, participants were assured—prior to submitting their narratives—that the data would be used exclusively for research purposes, that there were no “right” or “wrong” narratives, and that they should feel safe to express all perceptions, opinions, and feelings [13].

Convenience sampling was used, which may have engaged responses primarily from participants more actively involved in the project. This was acknowledged as a limitation.

The sample consisted of 24 students and 16 preceptors, totaling 40 individuals. Twenty-five participants completed the study, representing 65.5% of the sample. A total of 20 to 30 long texts are considered sufficient for analysis in IRaMuTeQ, provided the participant groups are homogeneous [15].

The results were discussed by an interprofessional group of three researchers—a nurse (MO), a physician (RK), and a biologist (FA)—each with prior experience in qualitative data analysis, which contributed to credibility and triangulation of perspectives. However, potential bias may have occurred. To address this, each researcher analyzed the narratives independently, followed by group discussions until consensus was reached. This process allowed the findings to be correlated with the analyses produced by the IRaMuTeQ software.

The narratives were originally written in Portuguese and translated into English by the research team. A double-checking procedure and consensus among researchers were used to ensure translation accuracy and semantic equivalence.

## Results

Twenty students and five preceptors involved in the program participated in the study. The Table 1 describes the sociodemographic and academic characteristics of the participants included in the study. Undergraduate programs refer exclusively to students (*n =* 20), whereas professional background refers exclusively to preceptors (*n =* 5).

**Table 1:** Main characteristics of the study population

Variable	n (%)
Sex	
Female	18 (72)
Male	7 (28)
Participant role	
Students	20 (80)
Preceptors	5 (20)
Undergraduate programs (students only)	
Physical Education	4 (20)
Psychology	4 (20)
Medicine	6 (30)
Nursing	3 (15)
Dentistry	3 (15)
Professional background (preceptors only)	
Psychologist	1 (20)
Physician	1 (20)
Nurse	1 (20)
Dentist	1 (20)
Nutritionist	1 (20)

The corpus consisted of 25 texts segmented into 321 text segments (TSs), of which 282 TSs (87.85%) were used. A total of 11,382 occurrences (words or word forms) emerged. The analyzed content was categorized into four classes:

- Class 1: “PET-Health as a Program that promotes Teamwork,” with 72 TSs (26.47%)
- Class 2: “Challenges Imposed by the Pandemic on the PET-Health Program,” with 48 TSs (17.64%)
- Class 3: “Adapting PET-Health Program to a New Reality,” with 95 TSs (34.93%)
- Class 4: “Teaching–Service Integration for the Development of Collaborative Competencies,” with 57 TSs (20.96%)

**Fig. 1:**
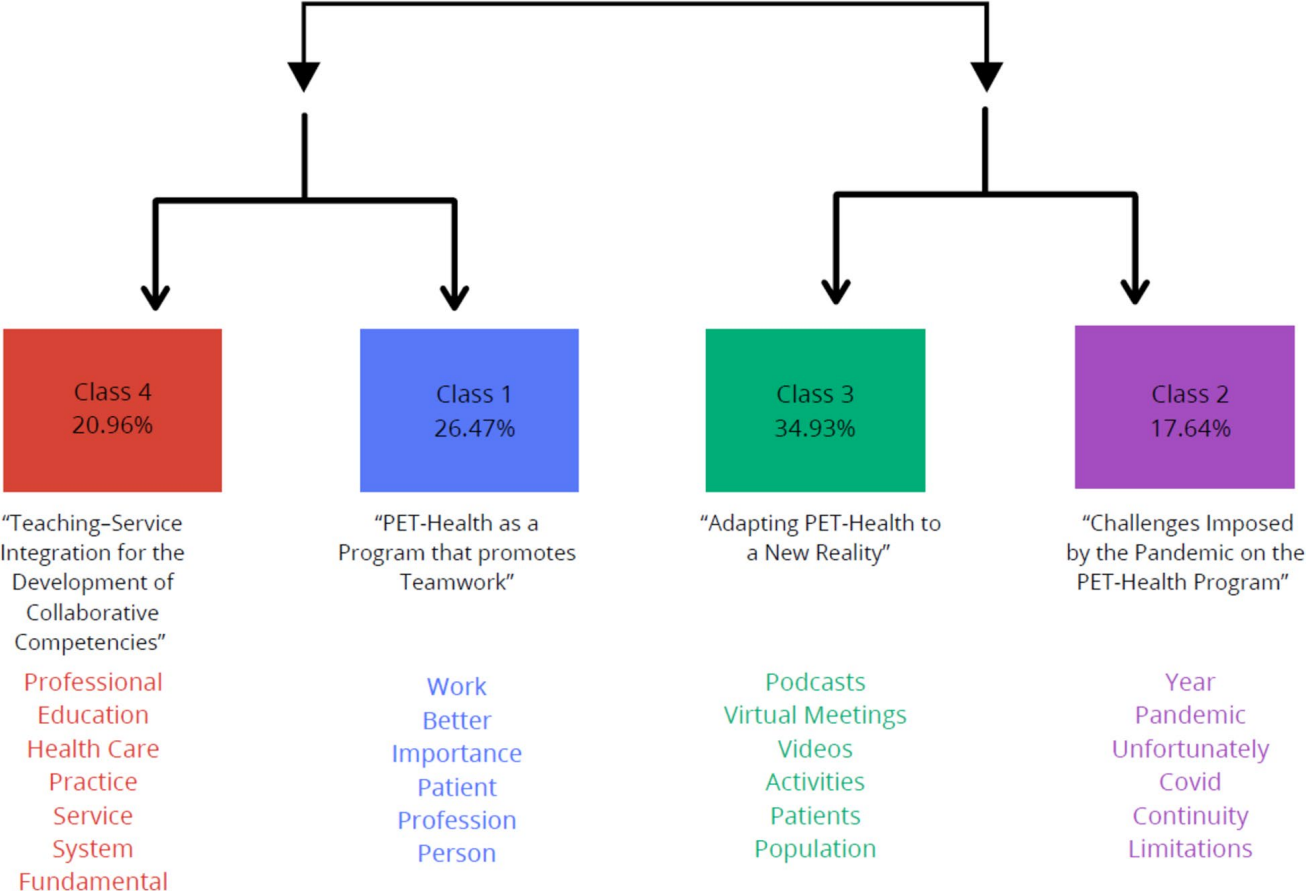
Dendrogram of descending hierarchical classification (IRaMuTeQ)

The Descending Hierarchical Classification (CHD) performed by IRaMuTeQ generated a dendrogram illustrating the lexical structure of the corpus. The dendrogram illustrates the division of text segments into four distinct lexical classes (Classes 1–4), generated through Descending Hierarchical Classification using IRaMuTeQ software. The classes were organized based on word co-occurrence and frequency, revealing thematic patterns emerging from participants’ narratives. The most frequent words associated with each lexical class are displayed in the figure (Fig. 1).

### Class 1

Comprised 26.47% (f = 72 TSs) of the analyzed corpus. This class included words such as “work” (χ^2^ = 23.39), “importance” (χ^2^ = 17.91), “patient” (χ^2^ = 14.40), “profession” (χ^2^ = 14.15), “multidisciplinary” (χ^2^ = 11.28), “future” (χ^2^ = 9.97), and “opinions” (χ^2^ = 8.43).

The content of this class was related to the learning opportunities provided by the PET-Health program, particularly regarding the development of collaborative competencies. It contained statements referring to enhanced understanding of professional roles and the relevance of each profession in patient care, as illustrated in the following excerpts:

“In this way, today I see myself as a professional student and a better person. Through the program, I was able to learn about the importance of integrating all areas in order to offer integrated care, and I was able to understand the importance of listening to and valuing others in teamwork.” (25).

“The results of this program are many and will forever be reflected in our way of working, always seeking the best for the patient and leaving aside the preconceptions embedded during our training, because each profession is important if we are to offer comprehensive, quality care to those who need it.” (22).

This class illustrates how interprofessional education strengthens and values each professional based on their specific roles, improves team communication, and develops the competencies required for collaborative practice.

### Class 2

Represented 17.64% (f = 48 TSs) of the analyzed corpus. This class included words such as “year” (χ^2^ = 72.97), “pandemic” (χ^2^ = 33.31), “unfortunately” (χ^2^ = 27.67), “COVID” (χ^2^ = 22.91), and “limitations” (χ^2^ = 18.22).

It reflected the difficulties experienced by the PET-Health program with the onset of the COVID-19 pandemic. The program was originally designed for interprofessional practice in the care of patients with chronic noncommunicable diseases in primary healthcare units. However, due to social distancing and the suspension of in-person academic activities, actions had to be adapted to ensure continuity. The following excerpts exemplify this experience:

“We had the program in full practical execution, then the pandemic came, and we had to adapt to a new virtual reality; a great challenge to continue the PET-Health program—it was a new experience.” (10).

“An immeasurable challenge to the continuity of the program was the imminence of the COVID-19 pandemic, as many activities had to be reformulated, virtual meetings were implemented, and the overload of services was reconciled.” (20).

These perceptions highlight the challenge imposed by the COVID-19 pandemic not only in healthcare, education, and public policy contexts but also in social domains, as corroborated by phenomenological perspectives.

### Class 3

Addressed adaptations of the UNIFAA PET-Health program following the declaration of the COVID-19 pandemic. It comprised 34.93% (f = 95 TSs) of the analyzed corpus. This class included words such as “podcasts” (χ^2^ = 19.34), “meetings” (χ^2^ = 15.81), “videos” (χ^2^ = 15.36), “population” (χ^2^ = 12.94), and “cordel” (a traditional form of Brazilian popular poetry based on rhymed and metrical verses) (χ^2^ = 9.49).

These activities were adapted to a remote model and referred to health interventions designed for each primary healthcare unit, based on the community health diagnoses conducted in the initial stage of the program. Interprofessional meetings and workshops were organized to enable the program’s continuity in a virtual environment. The following excerpts illustrate this experience:

“Through virtual meetings with Groups 1 and 2 of the PET-Health program, we began to devise strategies for continuing to bring improvements to the communities of Valença, even if only remotely, and so the preceptor began activities with the interprofessionality course.” (10).

“Group’s activity on social networks, producing podcast reports and taking part in informative talks broadcast to the community via local radio.” (17).

In Class 3, the technological disruption caused by COVID-19 in both education and healthcare is evident. Competencies such as creativity, connection, and, above all, collaboration were required to ensure the continuity of the project. However, social barriers became more evident, demonstrating that it is still not possible to provide education and healthcare universally.

### Class 4

Referred to the role of preceptors in the program, highlighting their experiences and mediating role between the territory and the educational institution. It comprised 20.96% (f = 52 TSs) of the analyzed corpus. This class included words such as “professionals” (χ^2^ = 44.19), “teaching” (χ^2^ = 31.09), “health” (χ^2^ = 26.89), and “practice” (χ^2^ = 22.20).

This class presented text segments related to the preceptors’ contributions to the educational process and their role as a link between the academic environment and health services. Primary healthcare units were identified as spaces where collaboration, communication, and quality of care could be observed. The following statements illustrate these contributions:

“Being able to observe brilliant professionals working as a team and being part of this work is undoubtedly the main thing, but also the contact with academics from other courses, with the users of the service, and with the dynamics of how the health units work.” (17).

“The receptiveness of the professionals and users in the primary care units, the collaborative work, the daily contact with the health network, the willingness of the students, and the many innovative methodologies used to conduct the groups are identified as facilitators for the success of the program.” (22).

Class 4 emphasizes the role of the preceptor as a mediator and mentor throughout the entire undergraduate journey. The construction of professional identity extends beyond the classroom to all health services where students gain experience during their academic training.

Table 2 describes the lexical classes, interpretive codes, and representative participant quotes derived from the IRaMuTeQ analysis.

**Table 2:** Analytical traceability between lexical classes and participant narratives

Class	Theme	Codes	Representative participant quotes
1	PET-Health as a program promoting teamwork	Teamwork; Collaboration; Comprehensive care; Interprofessional communication	“Every perspective from the different professions makes a difference to the patient. PET-Health changed my view and will make me a better professional in the future. It made me believe that sharing knowledge is possible and that the importance of each profession is equivalent.” (Participant 19) “The project is extremely important, as it makes us realize that working with professionals from other fields broadens our learning and allows us to understand different perspectives, not only from our own area but also from others.” (Participant 4) “The outcomes of this project are countless and will forever influence our way of working, driving us to always seek the best for patients and to overcome the preconceptions embedded in our training, since every profession is essential to provide comprehensive and high-quality care to those in need.” (Participant 11)
2	Challenges imposed by the COVID-19 pandemic	COVID-19; Adaptation; Disruption of activities	“However, the greatest challenge was the COVID-19 pandemic, as the project’s initial plan was to develop an action plan addressing users’ needs during the second year of activities. The preventive measures and social distancing requirements imposed by the pandemic did not allow this to be carried out as originally planned.” (Participant 5) “In the second year of the PET-Health program, we unfortunately faced the difficult situation experienced by the country and the world with the COVID-19 pandemic, which limited the scope of our work.” (Participant 1) “Thus, in-person activities were suspended, and we started to carry out everything remotely. Using the data collected during the first year of the project, we identified the critical issues in the neighbourhood and, together, developed possible intervention proposals.” (Participant 25)
3	Adaptation of the PET-Health program to virtual contexts	Health education; Health communication; Educational technologies	“We recorded videos and podcasts for the community, addressing the main topic and related issues, which were uploaded to the project’s platform, contributing even from a distance. During the same period, we held meetings and workshops that greatly enhanced our learning.” (Participant 12) “In 2020, while the situation was more favourable, we went to the Primary Health Care Unit to conduct surveys and identify the main myths and doubts of the population about the coronavirus. Subsequently, we used the information collected to guide the content we should produce in the form of texts, videos, and podcasts to be disseminated.” (Participant 20) “I believe that creating educational videos and podcasts was a good way we found to try to overcome this problem; however, the reach and the impact on lifestyle changes would have been greater with in-person activities.” (Participant 25)
4	Teaching–service integration and mentorship	Teaching–service integration; Mentorship; Community-based education	“In this context, preceptors act as bridges, mediating the relationship between educational institutions and health services. Their practical experience in public health enables students to integrate into this setting in a dynamic and up-to-date way.” (Participant 24) “Preceptors have practical experience in public health, which enables students to integrate into this context in a dynamic and up-to-date way. We act as intermediaries between educational institutions and the labour market.” (Participant 6) “Being able to observe brilliant professionals working as a team and to be part of that work is, without a doubt, the main highlight. But it also includes the contact with students from other courses, with service users, and with the dynamic functioning of the health units.” (Participant 17)

Lexical classes were derived from descending hierarchical classification using IRaMuTeQ software.Participant narratives were interpreted from a phenomenological perspective, focusing on lived experiences and meaning construction

## Discussion

The aim of this study was to assess the impact of the PET-Health program on the development of collaborative competencies through a qualitative investigation involving health students and preceptors.

Mapping conducted by the World Health Organization (WHO) on interprofessional education (IPE) practices worldwide has demonstrated benefits that converge with those found in this study [1].

Class 1 indicates that the PET-Health program fostered the development of collaborative competencies. The narratives reveal that participants perceived their lived experience as essential for improving communication and understanding professional roles within collaborative work, contributing to more integrated care for the community. From a phenomenological perspective, these findings indicate a re-signification of care practices toward a more comprehensive and resolutive approach, suggesting a transition from a uniprofessional mode of practice to an interprofessional collaborative logic. These perceptions are consistent with the literature, which highlights interprofessional activities as strategies associated with improvements in population health care and the strengthening of the health workforce [1, 5, 8, 17–19].

These experiences are aligned with the core competencies of collaborative practice—values and ethics, communication, teamwork, and roles and responsibilities—all directed toward improving individual and population health through continuous care [6].

Class 1 also highlights the Primary Health Care (PHC) setting. Participants perceived PHC as a privileged space for interprofessional learning and collaboration. This finding reinforces the existing literature, which emphasizes that PHC is not merely a practice setting but also a pedagogical space for developing collaborative competencies. Among the competencies potentially developed in this context are respect, awareness, appreciation of diversity, a focus on patient-centered care, greater understanding of the roles of other health professionals, increased synergy in team-based care, and improved patient care [20, 21].

Community-based programs contribute to reducing knowledge gaps, promoting collaborative leadership and communication skills, and fostering an understanding of professional roles in collaborative practice [22–24]. As an example, the Rosalind Franklin University of Medicine and Science (RFUMS) in the United States offers an IPE program with a strong community- and service-based curricular component. Students are required to work in teams to engage community partners in solving health problems, and the program has received positive evaluations from both students and community partners [22].

Class 2 reveals the experiences of participants in the PET-Health program during the COVID-19 pandemic. These findings suggest that this context was marked by uncertainty and distress, requiring the adaptation of previously planned health education and care activities to remote modalities. From a phenomenological perspective, this adaptation can be understood as a process of reconstructing meanings and collaborative practices in response to the disruption of the formative routine. Similar findings have been described in studies that analyzed adaptations of interprofessional programs during the COVID-19 pandemic [25, 26].

Class 3 highlights the adaptations undertaken by participants within the PET-Health program. The narratives indicate the potential of virtual environments for developing health education actions, including the production of podcasts, videos, and other strategies aimed at community care. However, participants also acknowledged that access to information revealed inequities in the field of health education. From a phenomenological perspective, these experiences indicate both the limits and possibilities of collaborative practice in virtual contexts, with these adaptations perceived as only partially effective. Similar findings have been reported in other studies, which emphasize that despite the obstacles and social distancing imposed by the pandemic, it was possible to develop successful health education actions, disseminate information, and maintain community ties established prior to the pandemic [25].

In the literature, even before the pandemic, virtual environments had already been proposed as platforms for implementing IPE programs. Nevertheless, these environments have not yet been sufficiently evaluated or fully explored. Moreover, IPE programs require robust infrastructure to support students, faculty, and health teams in virtual modalities [26].

Class 4 highlights the recognition of the preceptor in the PET-Health program as a central figure in the learning process and in mediating formative activities. Participants described the preceptor as playing a fundamental role in articulating teaching, service, and community. From a phenomenological perspective, these experiences indicate the role of the health professional as a mentor, integrating the teaching–learning process through practices lived in health services. These findings are consistent with studies that emphasize the workplace as a living space for training—settings where life unfolds and where competencies (knowledge, skills, and attitudes) inherent to health professions can be developed [27–29]. A systematic review reinforced these findings by identifying the quality of supervision as the most satisfying aspect reported during the development of IPE programs [30].

Faculty members also serve as role models for students, and their behaviors and attitudes tend to be mirrored by learners. In this context, experiencing interprofessional teamwork and having educators who believe in and practice collaboration are fundamental for the development of collaborative competencies [31]. Health educators—both professors and preceptors—should guide students in applying competencies acquired through collaborative practice in the workplace [31, 32].

Furthermore, Class 4 suggests that all students may benefit from opportunities to experience interprofessional education. Accordingly, the findings suggest that IPE could be longitudinal and intentionally embedded throughout health curricula, including research and community engagement activities [22–24]. In some countries, accreditation systems already require the incorporation of IPE into health curricula. In Brazil, the Accreditation System for Medical Schools (SAEME) considers interprofessional learning a quality indicator for medical education [32, 33].

For IPE to be effectively implemented, a clear conceptual framework and appropriate educational methods are essential. The vision for IPE must also be realistic, flexible, achievable, practical, focused, and transferable [34].

Implementing an interprofessional curriculum requires significant commitment and support from university leadership and faculty. Essential resources include engagement across multiple disciplines, dedicated time for planning and implementation, cross-curricular analysis, adequate infrastructure, appropriate methodologies to promote group cohesion, and reliable technological support [7, 31].

One limitation of this study was the limited ability to assess health outcomes resulting from PET-Health activities in the communities where tutorial groups operated. More rigorous studies are needed to evaluate the impact of interprofessional education on both health professionals and patient outcomes [8, 30, 35].

The PET-Health program promoted the implementation of interprofessional education through the integration of teaching, service, and community. Accordingly, the findings suggest that activities carried out in health care settings and their evaluation processes *could take into account* their effects on health outcomes [34]. Effective implementation of IPE requires investment in pedagogical models, faculty and preceptor development, financial resources, and institutional political support to ensure sustainability [1, 5, 6, 32, 34, 35]. Interprofessional education *is not intended to be* a one-time intervention but a continuous component of academic curricula, fostering a culture of collaboration.

This study has some limitations. Participants’ experiences were not compared across different academic programs; participant validation (member checking) was not conducted; and the analysis relied exclusively on IRaMuTeQ, which tends to emphasize dominant lexical structures and may limit the visibility of highly singular experiences or discordant positions that do not emerge as predominant lexical classes.

## Conclusions

Based on the narratives, the PET-Health program provided experiences of collaboration and supported the development of collaborative competencies, particularly by fostering respect for each profession. It was observed that the program also facilitated the recognition of professional roles, improved team communication, and reinforced patient-centered care. Based on the narratives, preceptors were highlighted as key professionals in integrating teaching, service, and community, as well as in fostering the development of collaborative competencies. They act as role models; when students experience teamwork in a concrete manner, they tend to reproduce these behaviors and attitudes.

The COVID-19 pandemic required the restructuring of the program into remote activities, which primarily focused on health education. This challenge, however, was addressed collectively, demonstrating that collaboration can contribute to overcoming barriers encountered in professional practice. The blended learning method, involving the community (primary health care), proved to be a positive strategy for the development of collaborative competencies, as students responded directly to the community’s complex and continually evolving needs. These needs reinforce the importance of teamwork in delivering effective health interventions.

In light of the findings of this study, interprofessional education is relevant to the training of health professionals and may be articulated with curriculum guidelines, pedagogical programs, and public health policies—particularly the National Policy on Permanent Health Education—as well as with the involvement of organized civil society and social representation. In this sense, interprofessional education may contribute to the construction of collaborative work environments and to the strengthening of patient safety.

## Supplementary Information

Supplementary Material 1.

Supplementary Material 2.

## Data Availability

The datasets generated during and/or analysed during the current study are available from the corresponding author upon reasonable request. Supplementary materials (COREQ checklist, dendrogram, and traceability table) are available online. Supplementary materials (COREQ checklist, dendrogram, and traceability table) are available online.

## Abbreviations

CHD: Descending Hierarchical Classification
COREQ: Consolidated Criteria for Reporting Qualitative Research
COVID-19: Coronavirus Disease 2019
IPE: Interprofessional Education
IRaMuTeQ: Interface de R pour les Analyses Multidimensionnelles de Textes et de Questionnaires
PET-Health: Education through Work for Health Program
PHC: Primary Health Care
SAEME: Accreditation System of Medical Schools
UNIFAA: Valença University Center
WHO: World Health Organization
