# Supplementary material for: Interprofessional education program: perceptions and experiences among Brazilian participants
Source: BMC Med Educ. 2026 Feb 24;26:569. doi: 10.1186/s12909-026-08810-x (PMC13063747; doi:10.1186/s12909-026-08810-x)
Supplement: Supplementary file 2 — Supplementary Material 2. [file 12909_2026_8810_MOESM2_ESM.docx]

**Appendix**

**Question**

Considering the lessons learned, limitations encountered, and observed changes in behaviour, how are you currently applying, and how do you plan to apply the skills acquired in the program your practice?
